# Lactate Transporter Monocarboxylate Transporter 4 Induces Bone Pain in Head and Neck Squamous Cell Carcinoma

**DOI:** 10.3390/ijms19113317

**Published:** 2018-10-25

**Authors:** Kazuaki Hasegawa, Tatsuo Okui, Tsuyoshi Shimo, Soichiro Ibaragi, Hotaka Kawai, Shoji Ryumon, Koji Kishimoto, Yuka Okusha, Nur Mohammad Monsur Hassan, Akira Sasaki

**Affiliations:** 1 Departments of Oral and Maxillofacial Surgery Okayama University Graduate School of Medicine, Dentistry, and Pharmaceutical Sciences, Okayama 700-8525, Japan; de421040@s.okayama-u.ac.jp (K.H.); shimotsu@hoku-iryo-u.ac.jp (T.S.); sibaragi@md.okayama-u.ac.jp (S.I.); de18018@s.okayama-u.ac.jp (H.K.); skutuku@yahoo.co.jp (S.R.); koujik@md.okayama-u.ac.jp (K.K.); aksasaki@md.okayama-u.ac.jp (A.S.); 2 Departments of Dental Pharmacology, Okayama University Graduate School of Medicine, Dentistry, and Pharmaceutical Sciences, Okayama 700-8525, Japan; okyu8m3m@hotmail.co.jp; 3 School of Dentistry & Health Sciences, Charles Sturt University, Sydney, NSW 2678, Australia; nhassan@csu.edu.au

**Keywords:** head and neck squamous cell carcinoma, bone pain, monocarboxylate transporter 4

## Abstract

Head and neck squamous cell carcinoma (HNSCC) poses a significant challenge clinically, as it can invade facial bones and cause bone pain that is undertreated and poorly understood. Here we studied HNSCC bone pain (HNSCC-BP) in an intratibial mouse xenograft model that uses a human HNSCC cell line (SAS cells). These mice develop HNSCC-BP associated with an upregulation of phosphorylated ERK1/2 (pERK1/2), which is a molecular indicator of neuron excitation in the dorsal root ganglia (DRGs) of sensory nerve cell bodies. Our experiments demonstrated that the inhibition of monocarboxylate transporter 4 (MCT4) by short hairpin (shRNA) transduction suppressed the HNSCC-BP, the lactate level in bone marrow, and the pERK1/2 expression in DRG. The sensory nerves also expressed increased levels of the acid-sensing receptor TRPV1. DRG neurons co-cultured with SAS cells showed increased neurite outgrowth, and were inhibited by MCT4 silencing with shRNA. Collectively, our results show that HNSCC induced an acidic bone microenvironment that evokes HNSCC-BP via MCT4 expression.

## 1. Introduction

Head and neck squamous cell carcinoma (HNSCC) frequently invades the facial bones [1], and this invasion promotes HNSCC bone pain (HNSCC-BP) [2]. Cancer pain contributes to increased mortality and is a prognostic factor of poor clinical outcomes [3]. HNSCC-BP often lowers a patient’s quality of life and leads to a disruption of speech and swallowing function; it thus poses a significant challenge to the quality of life of patients presenting with HNSCC-BP. The pathophysiology of bone pain associated with HNSCC is poorly understood, and HNSCC-BP is frequently inadequately treated.

Cancer cells have been demonstrated to secrete significant amounts of growth factors that can promote osteoclastogenesis [4,5]. Protons (H^+^) are well known as a pain inducer [6]. In a bone cancer microenvironment, cancer cells and osteoclasts secrete protons through the proton pump, creating an acidic microenvironment [7]. This environment breaks the bone matrix and activates pH-sensitive sensory neurons (SNs), eliciting bone pain [8]. It is thus critical to evaluate new approaches to the treatment of bone destruction and bone pain in advanced HNSCC. Monocarboxylate transporter 4 (MCT4), also known as SLC16A3, is a member of the MCT family. MCT4 is one the key transporters involved in the regulation of lactate and proton release from cancer cells [9]. MCT4 also plays a critical role in anaerobic glycolysis, known as the Warburg effect [10].

In normal cells, glucose is taken as an energy source. Glucose is converted to pyruvate in glycolysis pathway. Pyruvate is source of the mitochondrial oxidative glycolysis for ATP generation. In cancer cells glucose uptake and aerobic glycolysis is increased compared with normal cells [11]. A greater amount of glucose is diverted to biosynthetic pathways to fuel cell proliferation by anaerobic glycolysis. In this pathway, pyruvate is preferentially shunted to lactate, resulting in increased lactic acid production from cancer cells [12].

Lactic acid is considered an energy source in the central nervous system [13]. The effect of lactic acid on sensory nerve activation in HNSCC bone invasion is not yet known. We conducted the present study to evaluate the effect of MCT4 expression on HNSCC-BP and sensory neuron activation in bone. Our findings provide the first evidence that MCT4 has a role in HNSCC growth in bone, lactic acid release, and HNSCC-BP.

## 2. Results

### 2.1. MCT4 Expression in the Human HNSCC Samples

**Figure 1:**
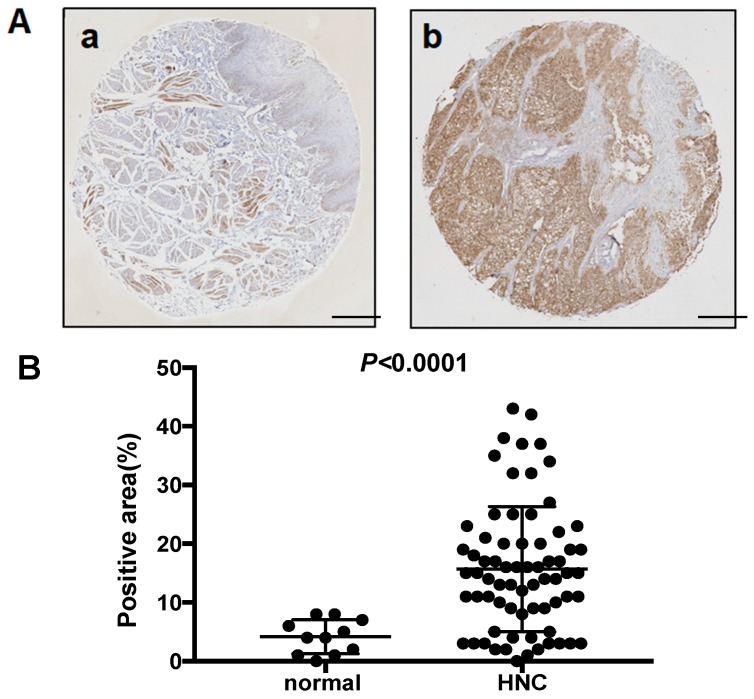

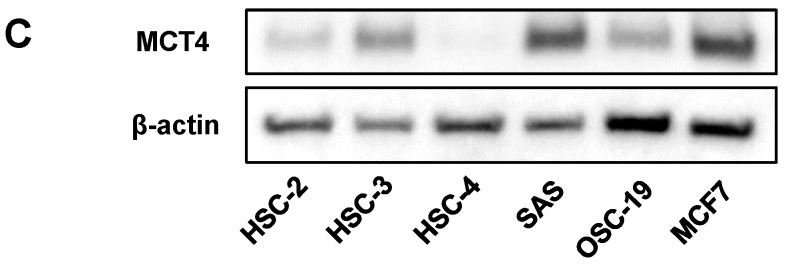
The expression of monocarboxylate transporter 4 (MCT4) in head and neck normal tissues, head and neck cancers, and human oral squamous cell lines. (**A**) Immunohistochemistry analysis of MCT4 in head and neck normal tissue (**a**) and head and neck cancer (HNC) (**b**). Scale Bar = 200 μm. (**B**) Scatterplot of the MCT4-positive areas in the head and neck normal tissues (*n* = 11) and head and neck squamous cell carcinoma (HNSCC) (*n* = 70). Error bars: mean ± standard deviation (SD). There was a significantly increased expression of MCT4 in the HNSCC samples (*p* < 0.0001). (**C**) Expression of MCT4 in human oral squamous cell carcinoma cell lines (HSC-2, -3, -4; SAS, OSC-19) and a human breast cancer cell line (MCF7) analyzed by western blotting.

Figure 1A provides a representative histologic pattern of normal oral tissue and HNSCC tissue. MCT4 was highly expressed in the HNSCC samples compared to the normal epithelium samples. The MCT4-positive area in each HNSCC sample indicated a significantly increased expression of MCT4 (*p* < 0.0001) (Figure 1A,B).

To determine whether oral squamous cell carcinoma cells express MCT4 in vitro, we performed a western blot analysis of MCT4 expression in the HNSCC cell lines HSC-2, HSC-3, HSC-4, SAS, and OSC-19, and in MCF-7 breast cancer cells. As shown in Figure 1C, the results of the western blot analysis revealed a high expression of MCT4 in the SAS cells. 

### 2.2. The Reduction of MCT4 Resulted in Decreased Lactic Acid Release

**Figure 2:**
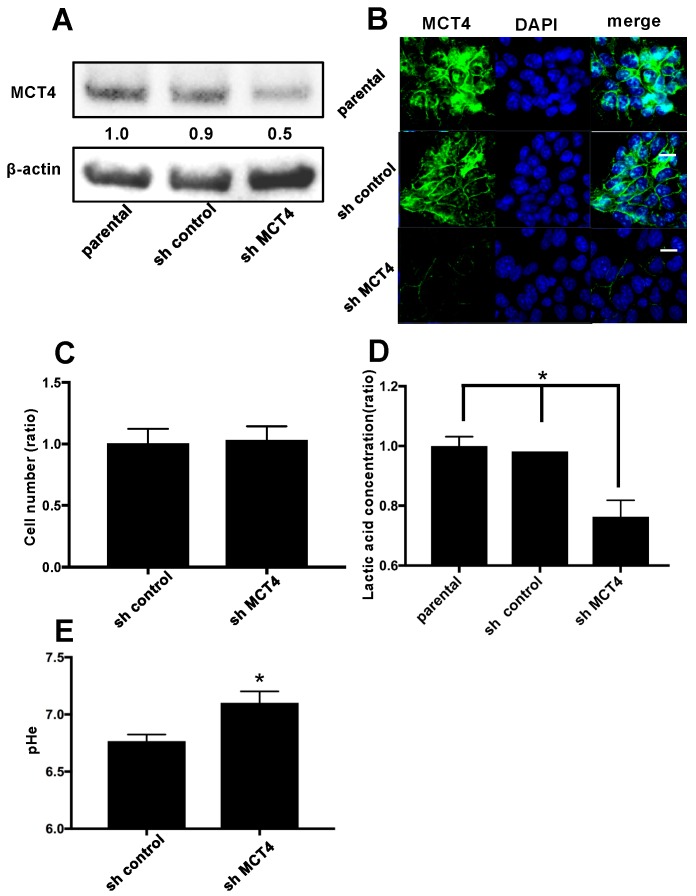
Reduction of MCT4 protein in HNSCC SAS cells. (**A**) The expression of MCT4 in SAS cells. After the stable transfection of SAS cells with control shRNA (sh-control) and MCT4 shRNA (sh-MCT4), the transfected and non-transfected cells (parental) were analyzed by western blotting. The expression of MCT4 in sh-MCT4 analyzed with image blot density was approximately one-half that of the parental cells. (**B**) Immunofluorescence analysis of MCT4 and 4′,6-diamidino-2-phenylindole (DAPI) for nuclear staining in the cells. Left, MCT4 (green); middle, DAPI (blue); right, merge. Sections were incubated with rabbit anti-MCT4 (1:100), then with Alexa Fluor 488 anti-rabbit IgG (1:1000) and encapsulated with DAPI. Scale Bar = 100 μm. (**C**) Proliferation assay. We cultured sh-control and sh-MCT4 cells for seven days and then counted the number of each (*n* = 3). There was no significant difference between the cells. Error bars: mean ± SD. (**D**) Each group of SAS cells was cultured for 24 h. The conditioned medium was then collected and the concentration of lactic acid was measured (*n* = 3). Error bars: mean ± SD; * *p* < 0.01 vs. sh-MCT4. (**E**) Each group of SAS cells was cultured for 48 h. The conditioned medium was then collected and the extra cellular pH (pH_e_) was measured (*n* = 3). Error bars: mean ± SD; * *p* < 0.01 vs. sh-MCT4.

To examine the role of MCT4 in HNSCC, we introduced a shRNA plasmid targeting MCT4 into SAS cells by means of an electroporation system. As shown in Figure 2A, the expression of MCT4 protein was 40–50% suppressed in the sh-MCT4-transfected groups compared to the parental SAS cells and control shRNA (sh-control) plasmid-introduced group.

Figure 2B provides the results of immunofluorescence cytochemistry staining. The membrane-bound MCT4 expression was decreased in the MCT4-knockdown SAS cells compared to the parental SAS cells. The proliferation of MCT4-knockdown SAS cells did not differ significantly from that of the sh-control SAS cells (Figure 2C). However, the lactic acid efflux and lowering of the extra cellular pH (pH_e_) were suppressed by 40% compared to those in the parental and control shRNA-introduced SAS cells (Figure 2D,E). 

### 2.3. The Reduction of MCT4 in SAS Cells Decreased Sensory Nerve Sprouting

**Figure 3:**
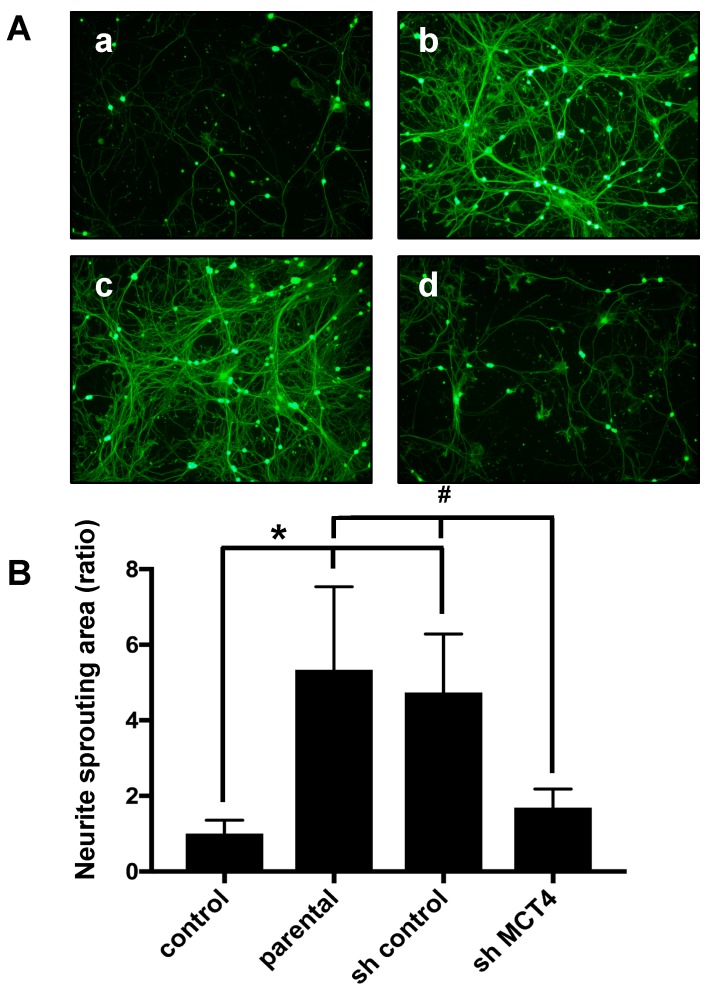
Neurite outgrowth from rat primary dorsal root ganglia (DRG) cells co-cultured with each group of SAS cells. (**A**) Neurite outgrowth from primary neuron cells in co-culture with control medium (**a**), parental SAS (**b**), sh-control (**c**), and sh-MCT4 (**d**) SAS cells for five days, labeled with calcein acetoxymethyl (AM). Scale Bar=50μm. (**B**) Quantitative data of (**A)**. Error bars: mean ± SD; * = *p* < 0.05 vs. control; # = *p* < 0.05 vs. sh-MCT4.

To analyze the effects of MCT4 reduction in SAS cells on SN fiber sprouting, we co-cultured primary sensory neuron cells with MCT4-knockdown SAS cells for five days and evaluated the neurite outgrowth, which is an in vitro indicator of sprouting. We observed that the neurite outgrowth of the primary neuron cells was increased by co-culturing with parental SAS or control shRNA SAS cells (Figure 3A(b,c)). Conversely, the co-culturing with MCT4-knockdown SAS cells significantly decreased the neurite sprouting (Figure 3A(d)). The neuron areas were evaluated with ImageJ software (U.S. National Institutes of Health). As shown in Figure 3B, the parental and sh-control SAS cells exhibited significantly increased neurite outgrowth, and the MCT4 knockdown suppressed the neurite outgrowth. 

### 2.4. MCT4 Expression and HNSCC-BP Were Associated with SAS-Colonized bone

**Figure 4:**
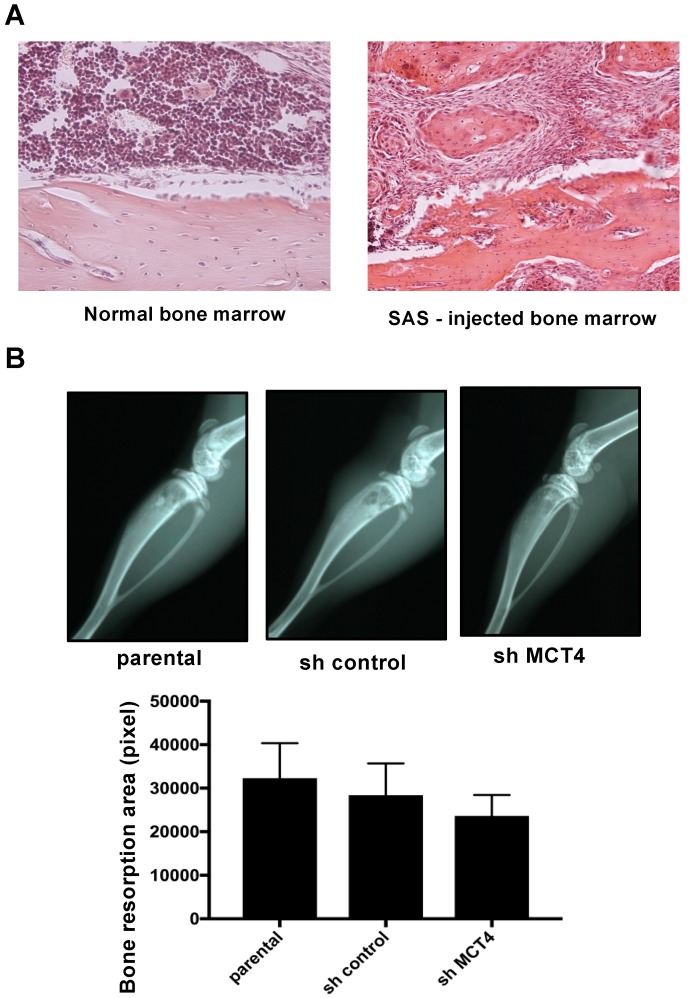
Bone resorption of the tibia in SAS cell-injected mice. (**A**) Normal bone marrow tissue and SAS cells colonized in bone marrow (hematoxylin and eosin) at 100× magnification. (**B**) X-ray photographs showing bone resorptions of the tibia at post-operative day (POD) seven in SAS cell-injected mice. The lower panel compares the bone resorption areas. There was no significant difference between the parental and sh-MCT4 groups (*n* = 5). Error bars: mean ± SD.

The mice that were intratibially injected with SAS cells developed aggressive proliferation of SAS cells in the bone marrow and bone destruction as revealed by X-ray (Figure 4A,B). The bone destruction areas were visualized as radiographic resorption lesions. There was no significant difference in destruction area between parental, sh-control and sh-MCT4 SAS-injected mice (Figure 4B).

**Figure 5:**
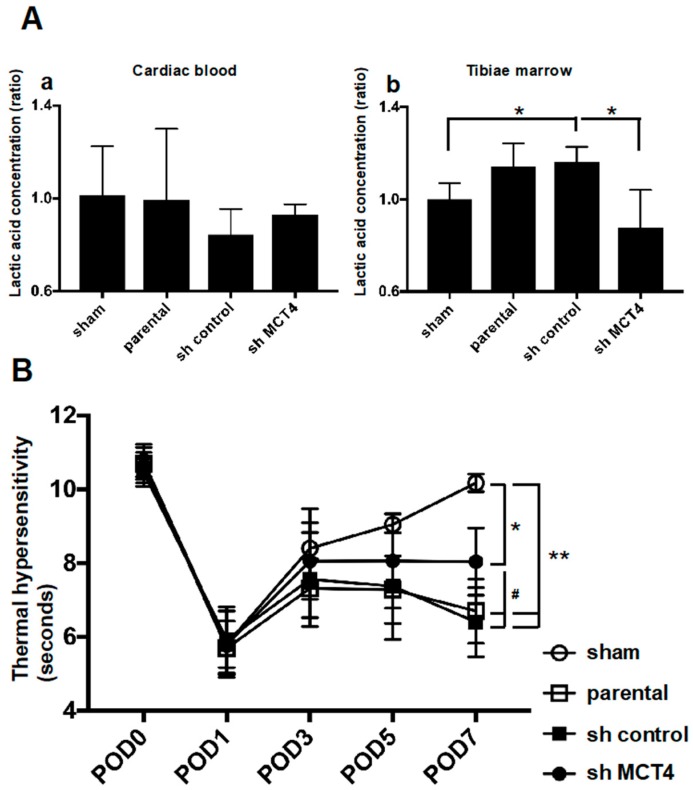
Bone pain and lactate concentration in sham mice and SAS cell-injected mice. (**A**) Lactate concentration in cardiac blood sampling or tibiae marrow (*n* = 5). (**a**) Lactic acid concentration in cardiac blood. (**b**) Lactic acid concentration in tibial bone marrow. Error bars: mean ± SD. * = *p* < 0.05 vs. sh-control. (**B**) Thermal sensitivity results of each group. The test was performed every other day from POD one to seven (*n* = 5). Error bars: mean ± SD. * = *p* < 0.05 vs. sham; ** = *p* < 0.01 vs. sham; # = *p* < 0.05 vs. sh-MCT4.

To evaluate the direct effect of MCT4 expression in SAS-induced HNSCC-BP, we investigated SAS injection-induced thermal hyperalgesia and the lactic acid concentration in bone marrow after the injection of SAS cells. Injection of the cancer into the tibia did not affect the systemic lactic acid concentration (Figure 5A(a)). In contrast, the lactic acid concentration in bone marrow was significantly increased by the SAS cell injection into bone marrow. Importantly, MCT4-knockdown SAS cells colonized in bone significantly inhibited the lactic acid concentration (Figure 5A(b)). In parallel with the bone marrow lactic acid concentration, the progression of allodynia and thermal hyperalgesia was significantly suppressed by MCT4 knockdown in SAS cells seven days after the cancer cell injection (Figure 5B).

**Figure 6:**
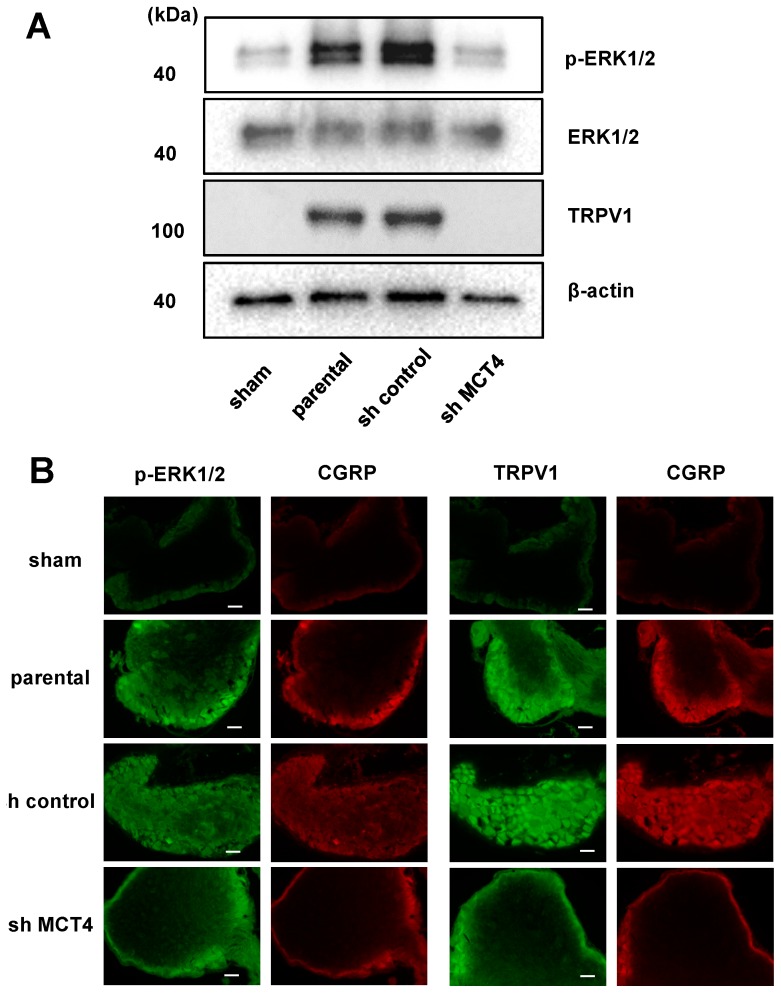
Excitation of sensory nerves determined by p-ERK expression in DRGs in sham, parental, sh-control, and sh-MCT4-injected mice shown by TRPV1 expression. (**A**) Western blotting results. (**B**) Immunofluorescence analysis of p-ERK and TRPV1. (**left**) p-ERK or TRPV1 (green); (**right**) CGRP (red). Scale Bar = 50 μm. Sections were processed as described in the Materials and Methods section.

In parallel with the HNSCC-BP results, the DRGs from the SAS cell-injected mice demonstrated an increased expression of ERK1/2 phosphorylation, which is a molecular indicator of neuron excitation [14]. The DRGs from MCT4-knockdown SAS cell-injected mice showed decreased pERK1/2 expression in the western blot analysis (Figure 6A). We previously reported that an acidic microenvironment increased the expression of acid-sensing receptor Acid-sensing ion channel 3 (ASIC3) in sensory neurons [14]. In the present study, we evaluated whether the expression of another acid-sensing receptor (TRPV1) in the DRGs of sensory neurons is increased by lactic acid. The western blotting analysis revealed that TRPV1 expression in DRGs was significantly increased by the SAS cell injection in tibias, whereas the injection of MCT4-knockdown SAS cells decreased the TRPV1 expression compared to those in the parental and control shRNA-transduced SAS cells (Figure 6A). These results demonstrated that MCT4 knockdown decreased pERK1/2 and TRPV1 upregulation in DRGs by SAS injection into the tibia, as shown by the immunofluorescence staining (Figure 6B).

## 3. Discussion

MCTs are part of a 14-member family of transporter proteins [15]. The first four isoforms (MCT1–4) have a function in transport proton-linked carbohydrates such as pyruvate, lactate, and ketone bodies [16]. MCT4 contributes to the maintenance of intracellular pH within the physiological range. The maintenance of pH preserves metabolism, protein synthesis, and signaling processes.

MCT4 mediates the lactic acid efflux from cells that are dependent on glycolysis for their ATP production. MCT4 expression is limited to skeletal muscle, red blood cells, brain tissue and tumor cells that are highly glycolytic tissues [17,18]. The metabolic feature of malignant tumor is anaerobic glycolysis which produces huge amount of lactic acid. Cancer cells discharge lactic acid to evade intracellular acidification [10]. Thus, it seems reasonable that malignant tumor cells expressed MCTs. 

Our present findings indicate that SAS cells express plasma membrane MCT4, which acidifies the extracellular environment by releasing protons and directly contributes to the activation of sensory neurons to induce HNSCC-BP.

Our experiments revealed that tissues from patients with HNSCC expressed high levels of MCT4 compared to normal oral tissue. We thus evaluated the expression of MCT4 in several HNSCC cell lines and a positive control breast cancer cell line, MCF-7. We then investigated the effect of MCT4 on the lactic acid efflux in the HNSCC cell lines. We created MCT4-knockdown HNSCC SAS cells with shRNA transduction methods, and we observed that a 50% MCT4 protein reduction in the cell membrane decreased the lactic efflux in SAS cells, even though MCT4 expression did not affect cell proliferation. It was reported that MCT4 reduction decreased cancer cell proliferation in vitro [19], and in another study, HNSCC cells highly expressed the B subunit of lactate dehydrogenase enzyme (LDH-B), which converts lactate to pyruvate in aerobic glycolysis [20]. This aerobic metabolism may promote sh-MCT4 SAS cell growth in the same way as in parental SAS cells under in vitro culture conditions.

The best-studied roles of lactic acid are those in the central nervous system and neuron energy [21]. The effect of lactic acid on sensory neurons is unclear. We demonstrated in an earlier study that vacuolar-ATPase expressed in tumor cells decreased the tissue pH and stimulated both neurite sprouting and cancer bone pain [14]. The pKa value of lactic acid is 3.86, and thus lactic acid efflux decreases the pH [22]. Our present findings demonstrate that: (1) sensory neuron fiber sprouting was significantly increased in co-culture with an HNSCC cell line, and (2) MCT4 knockdown in HNSCC cells decreased the fiber sprouting. These data support our hypothesis that lactic acid also provides nutrition and induces sensory neuron axis sprouting.

Here, the mice that were intratibially injected with SAS cells developed osteolytic lesions as seen by X-ray (Figure 4B). These lesions showed an aggressive proliferation of SAS cells in the bone marrow (Figure 4A). The right legs of the mice harboring SAS cells initially displayed thermal hypersensitivity, and these pain behaviors progressed in parallel with the bone destruction, demonstrating that HNSCC-BP is associated with local SAS colonization (Figure 4 and Figure 5B).

It has been indicated that neurons promote cancer progression [23]. In the present study, the reduction of MCT4 did not affect cell proliferation. However, MCT4 knockdown decreased HNSCC-BP in mice. These results may have been caused by a lactic acid-induced activation of neurons and other bone micro-environmental cells that promoted HNSCC-BP.

Our results showed that MCT4 reduction inhibited the lactic acid concentration in both bone marrow and HNSCC-BP. Yoneda demonstrated that an acidic micro-environment created by osteoclasts and cancer in bone induces sensory excitation and evokes bone pain [24]. The results of our present investigation demonstrated that MCT4 expression in HNSCC cells and lactic acid efflux are key factors for HNSCC-BP.

Neuron cell excitation by protons was caused by an acid-sensing receptor. TRPV1 and ASIC3 are well-known acid-sensing receptors that are expressed in sensory neuron fibers. Wakabayashi showed that the disruption of the TRPV1 gene attenuated cancer-induced bone pain [6]. We propose that the low pH of HNSCC-colonized bone induces the activation of ASIC3, TRPV1, or both on pH-sensitive sensory neurons to induce HNSCC-BP. Our present data demonstrate that MCT4-derived lactic acid increased and activated the acid-sensing receptor TRPV1.

Recent studies reported that the tissue damage and inflammation caused by cancer greatly decreases the thresholds of TRPV1 and ASIC3 for sensing noxious stimulation [14]. We therefore speculate that lactic acid from cancer cells promotes HNSCC-BP in a vicious cycle via acid-sensing receptor activation and decreased thresholds for sensing noxious stimuli.

In conclusion, our present findings demonstrate that the creation of acidic extracellular bone micro-environments by HNSCC via lactic acid production through plasma membrane MCT4 and the responses of the sensory neurons innervating bone to the acidic microenvironment via TRPV1 are critical contributors to the pathophysiology of HNSCC-BP. Targeting these pathways may provide effective mechanism-based therapies for the control of HNSCC bone pain, which is currently undertreated.

## 4. Materials and Methods

### 4.1. Reagents

Puromycin dihydrochloride (cat. No. #sc-108071), control shRNA plasmid-A (#sc108060), MCT4 shRNA plasmid (h2) (#sc-45892-SH) and anti-MCT4 antibody (anti-rabbit, monoclonal, #sc-50329) were purchased from Santa Cruz Biotechnology (Dallas, TX). Anti-phospho-p44/42 MAPK antibody (p-ERK; anti-rabbit, monoclonal, #4370), anti-p44/42 MAPK antibody (ERK; anti-rabbit, monoclonal, #4695), horseradish peroxidase (HRP)-conjugated IgG antibody (goat anti-rabbit, monoclonal, #7074), HRP-conjugated IgG antibody (goat anti-mouse, monoclonal, #7076), Alexa Fluor 488-conjugated IgG (H+L) F(ab′)2 fragment (goat anti-rabbit, monoclonal, #4412), and Alexa Fluor 488-conjugated IgG (H+L) F(ab′)2 fragment (goat anti-mouse, monoclonal, #4408) were purchased from Cell Signaling Technology (Danvers, MA). Anti-VR1 antibody (TRPV1; anti-mouse, monoclonal, #ab203103), anti-CGRP antibody (anti-goat, polyclonal, #ab36001) and Alexa Fluor 647-conjugated IgG H&L (donkey anti-goat, monoclonal, #ab150135) were purchased from Abcam (Cambridge, MA, USA).

### 4.2. Cell Lines and Culture Conditions

The human oral squamous cell carcinoma cell lines SAS (#JCRB0260), HSC-2 (#JCRB0622), HSC-3 (#JCRB0623), HSC-4 (#JCRB0624), OSC-19 (#JCRB0198), and MCF-7(#JCRB0134) were obtained from the Human Science Resources Bank (Osaka, Japan). They were cultured in Dulbecco’s modified Eagle’s medium (DMEM) (Thermo Fisher Scientific, Waltham, MA, USA) supplemented with 10% heat-inactivated fetal bovine serum (FBS) and 1% penicillin–streptomycin in an atmosphere of 5% CO_2_ at 37 °C.

Rat DRG cells were obtained from Cell Applications (San Diego, CA, USA) and cultured according to the supplier’s instructions.

Cells (1 × 10^5^/96-well) were cultured for 48 h and extracellular pH (pHe) was measured by using a FiveEasy pH meter (Metter Toled) immediately after removal from the CO_2_ incubator.

### 4.3. Immunohistochemical Analysis

We analyzed the expression of MCT4 in head and neck cancer tissue and a normal tissue microarray (#HN803d; US Biomax, Rockville, MD, USA). The antigen was activated by cooking in a citric acid solution. For the immunohistochemical analysis, the specimens were incubated with anti-MCT4 antibody (1:100) overnight at 4 °C. The slides were then treated with a streptavidin–biotin complex (EnVision System labeled polymer, HRP; Dako, Carpinteria, CA, USA) for 60 min at a dilution of 1:100. The immunoreaction was visualized with the use of a DAB substrate–chromogen solution (Dako Cytomation Liquid DAB Substrate Chromogen System, Dako, Carpinteria, CA, USA). Quantification was performed using a BZ-X800 Analyzer hybrid cell count system (Keyence, Osaka, Japan), and the relative integrated density was calculated.

### 4.4. Analysis of MCT4 Expression in SAS Cells

SAS cells were transfected with 1 µg control short hairpin (sh) RNA or MCT4 shRNA with the use of 4D-Nucleofector™ (Lonza, Tokyo, Japan). Two days later, the cells were cultured in DMEM plus 10% FBS for 5 days in the presence of 2 µg/mL puromycin dihydrochloride for the selection of cells that stably expressed the shRNAs.

MCT4 shRNA plasmid is a pool of three different shRNAs. The hairpin sequence is as follows: GATCCGTCTACCTCTTCAGCTTCTTTCAAGAGAAGAAGCTGAAGAGGTAGACTTTTT. The corresponding siRNA sequences are 1: Sense: GUCUACCUCUUCAGCUUCUtt. Antisense: AGAAGCUGAAGAGGUAGACtt. The corresponding siRNA sequences are 2: Sense: GUCUACAUGUACGUGUUCAtt. Antisense: UGAACACGUACAUGUAGACtt. The corresponding siRNA sequences are 3: Sense: GUCAUUCCAGAGUGGAUCUtt. Antisense: AGAUCCACUCUGGAAUGACtt. All sequences are provided in 5′ → 3′ orientation.

### 4.5. Cell Proliferation Assay

SAS cells were plated in six-well plates at 1 × 10^5^ cells per well. Their number was counted 72 h later with a TC20 automated cell counter (Bio-Rad, Hercules, CA, USA).

### 4.6. Western Blot Analysis

The cell lysates were mixed with 4× Laemmli sample buffer (Bio-Rad) and heated at 95 °C for 5 min. The samples were electrophoresed in 4–12% SDS-PAGE gels, and the proteins were transferred onto PVDF membranes (Bio-Rad). The membranes were incubated with primary and secondary antibodies according to the ECL chemiluminescence protocol (RPN2109; Amersham Biosciences, Buckinghamshire, UK) to detect secondary antibody binding. Antibodies against MCT4 (1:1000), p-Erk (1:1000), Erk (1:1000) and TRPV1 (1:1000) were used as a primary antibody. HRP-conjugated anti-rabbit antibody (1:2000) and HRP-conjugated anti-mouse antibody (1:2000) were used as the secondary antibody. A BZ-X microscope (Keyence) was used for the analysis of western blots.

### 4.7. DRG Fiber Sprouting Assay

DRG cells were plated in 48-well plates (1 × 10^4^/well) with neuron growth media for 24 h. Subsequently, the DRG cells were co-cultured with SAS, short hairpin (sh) control SAS, and sh-MCT4 SAS in the transwell chamber for 5 days. DRG fibers were visualized with calcein acetoxymethyl (AM) staining. DRG neuron fibers were observed with an Olympus microscope (IX71; Tokyo, Japan).

### 4.8. Animal Experiments

We established a mouse model of bone invasion by human oral squamous cell carcinoma in 5-week-old female BALB/c nude mice (each group, *n* = 5; total, *n* = 20; mean body weight, 19.5 g; Charles River Laboratories, Yokohama, Japan) by the inoculation of 1 × 10^5^ SAS cells (parental, sh-control, sh-MCT4) with a 29-gauge needle into the bone marrow space of the right tibial edge of the mouse under anesthesia with 0.4 mg/kg of medetomidine, 4.0 mg/kg of midazolam and 5.0 mg/kg of butorphanol. The sham procedure was only a puncture with a 29-gauage needle into the right tibial cavity.

Thermal hyperalgesia and allodynia were evaluated every other day from POD 1–7 in each group of mice, respectively [25]. The mice were individually placed in a cage with a glass floor, over a moveable infrared light source. The light source was positioned under the center of the hind paw, and the time (seconds) from stimulus onset to withdrawal was recorded. On POD 8, the mice underwent cardiac blood collection under anesthesia, followed by cervical dislocation. Ipsilateral DRGs and the right tibia were then harvested. All of the animal experimental protocols were approved by the Ethics Review Committee for Animal Experimentation of the Okayama University Graduate School of Medicine and Dentistry (OKU-2013108, 19/Feb/2016. OKU-2018510, 7/May/2018).

### 4.9. In Vivo Analysis of HNSCC-BP 

We evaluated the thermal hypersensitivity and allodynia in the mice with the use of a Hargreaves apparatus (Plantar Test, #37370; Ugo Basile, Varese, Italy), which measures an animal’s withdrawal latency from a radiant heat source directed at the proximal half of the plantar surface of the ipsilateral hind paw. Prior to this examination, the mice were allowed to acclimate for 30 min to the testing environment, which consisted of translucent plastic-walled individual chambers and a 3-mm thick glass bottom. A radiant heat source, consisting of an adjustable infrared lamp and a built-in stopwatch accurate to 0.1 second, was used to measure the paw withdrawal latency. The test was performed every other day from POD 1–7 in each group.

### 4.10. In Vivo Radiography and Measurement of Osteolytic Lesion Areas

Osteolytic bone destruction was assessed on radiographs. The bones were placed against films (22 × 27 cm; Fuji Industrial Film FR: Fuji Photo Film Co., Tokyo, Japan) and exposed to soft X-rays at 35 kV for 15 seconds with the use of a Sofron apparatus (Sofron, Tokyo, Japan). The radiolucent bone lesions were observed microscopically (IX81, Olympus), and the areas were quantified with Lumina Vision/OL (Mitani, Tokyo).

### 4.11. Lactate Concentration Measurement

The lactic metabolism of tibias collected for a previous experiment was evaluated. Both ends of the tibias were cut, and the bone marrow serum was extracted by centrifugation. The lactate concentrations in the bone marrow serum and whole blood serum were measured by a lactate assay kit (EnzyChrom™; BioAssay Systems, Hayward, CA, USA).

### 4.12. DRG Processing

Corrected DRGs were homogenized in RIPA lysis buffer with 1 mM PMSF and phosphatase inhibitor (Na_3_VO_4_ and NaF) added. The lysate was centrifuged at 15,000× *g* for 5 min at 4 °C, and the supernatant was collected as total protein. Some of the collected DRGs were fixed in 10% neutral-buffered formalin and then embedded in paraffin. Western blotting and immunofluorescence were performed using these DRGs.

### 4.13. Immunofluorescence Analysis

We conducted an immunofluorescence analysis to determine the expressions of p-Erk and TRPV1 in DRGs from each group of mice. The specimens were incubated with 3% bovine serum albumin–phosphate buffered saline (BSA–PBS) blocking solution, and then with p-Erk antibody (1:200) or TRPV1 antibody (1:100) and anti-CGRP antibody (1:200) overnight at 4 °C as primary antibodies, followed by Alexa Fluor 488 anti-rabbit IgG (1:1000) or Alexa Fluor 488 anti-mouse IgG (1:1000) and Alexa Fluor 647 anti-goat IgG (1:1000) as secondary antibodies. Nuclei were counterstained with Fluoroshield mounting medium with DAPI (#ab104139; Abcam).

### 4.14. Statistical Analyses

We analyzed the data using an unpaired Student’s *t*-test for comparisons of two groups and by performing a one-way analysis of variance (ANOVA) and a post hoc Bonferroni or Dunnett test for the analysis of multiple group comparisons, using SPSS statistical software, ver. 10. Results are expressed as the mean ± standard deviation (SD). Probability (*p*) values < 0.05 were considered significant.
